# Association of demographics, HCV co‐infection, HIV‐1 subtypes and genetic clustering with late HIV diagnosis: a retrospective analysis from the Japanese Drug Resistance HIV‐1 Surveillance Network

**DOI:** 10.1002/jia2.26086

**Published:** 2023-05-23

**Authors:** Machiko Otani, Teiichiro Shiino, Atsuko Hachiya, Hiroyuki Gatanaga, Dai Watanabe, Rumi Minami, Masako Nishizawa, Takanori Teshima, Shigeru Yoshida, Toshihiro Ito, Tsunefusa Hayashida, Michiko Koga, Mami Nagashima, Kenji Sadamasu, Makiko Kondo, Shingo Kato, Shunsuke Uno, Toshibumi Taniguchi, Hidetoshi Igari, Sei Samukawa, Hideaki Nakajima, Yusuke Yoshino, Masahide Horiba, Hiroshi Moro, Tamayo Watanabe, Mayumi Imahashi, Yoshiyuki Yokomaku, Haruyo Mori, Teruhisa Fujii, Kiyonori Takada, Asako Nakamura, Hideta Nakamura, Masao Tateyama, Shuzo Matsushita, Kazuhisa Yoshimura, Wataru Sugiura, Tetsuro Matano, Tadashi Kikuchi, Japanese Drug Resistance HIV‐1 Surveillance Network

**Affiliations:** ^1^ AIDS Research Center National Institute of Infectious Diseases Tokyo Japan; ^2^ Institute of Medical Science University of Tokyo Tokyo Japan; ^3^ Center for Clinical Sciences National Center for Global Health and Medicine Tokyo Japan; ^4^ Clinical Research Center National Hospital Organization Nagoya Medical Center Aichi Japan; ^5^ Department of Laboratory Medicine Tokyo Medical University Tokyo Japan; ^6^ Department of Clinical Laboratory Sciences Nitobe Bunka College Tokyo Japan; ^7^ AIDS Clinical Center National Center for Global Health and Medicine Tokyo Japan; ^8^ AIDS Medical Center National Hospital Organization Osaka National Hospital Osaka Japan; ^9^ Internal Medicine Clinical Research Institute National Hospital Organization Kyushu Medical Center Fukuoka Japan; ^10^ Department of Clinical Laboratories Hokkaido University Hospital Hokkaido Japan; ^11^ Department of Hematology, Faculty of Medicine Hokkaido University Hokkaido Japan; ^12^ School of Medical Technology Health Sciences University of Hokkaido Hokkaido Japan; ^13^ Department of Infectious Diseases National Hospital Organization Sendai Medical Center Miyagi Japan; ^14^ Tokyo Metropolitan Institute of Public Health Tokyo Japan; ^15^ Division of Microbiology Kanagawa Prefectural Institute of Public Health Kanagawa Japan; ^16^ Department of Infectious Diseases Keio University School of Medicine Tokyo Japan; ^17^ Department of Infection Control Chiba University Hospital Chiba Japan; ^18^ Department of Hematology and Clinical Immunology Yokohama City University School of Medicine Kanagawa Japan; ^19^ Department of Microbiology Teikyo University School of Medicine Tokyo Japan; ^20^ Department of Respiratory Medicine National Hospital Organization Higashisaitama National Hospital Saitama Japan; ^21^ Department of Respiratory Medicine and Infectious Diseases Niigata University Graduate School of Medical and Dental Sciences Niigata Japan; ^22^ Department of Immunology and Infectious Disease Ishikawa Prefectural Central Hospital Ishikawa Japan; ^23^ Division of Microbiology Osaka Institute of Public Health Osaka Japan; ^24^ Division of Transfusion Medicine Hiroshima University Hospital Hiroshima Japan; ^25^ Postgraduate Clinical Training Center Ehime University Hospital Ehime Japan; ^26^ Division of Virology Fukuoka Institute of Health and Environmental Sciences Fukuoka Japan; ^27^ Department of Infectious, Respiratory, and Digestive Medicine, Control and Prevention of Infectious Diseases, Graduate School of Medicine University of the Ryukyus Okinawa Japan; ^28^ Joint Research Center for Human Retrovirus Infection Kumamoto University Kumamoto Japan

**Keywords:** CD4 counts, hepatitis C, HIV, Japan, late diagnosis, phylogenetic clustering

## Abstract

**Introduction:**

Late diagnosis of the human immunodeficiency virus (HIV) is a major concern epidemiologically, socially and for national healthcare systems. Although the association of certain demographics with late HIV diagnosis has been reported in several studies, the association of other factors, including clinical and phylogenetic factors, remains unclear. In the present study, we conducted a nationwide analysis to explore the association of demographics, clinical factors, HIV‐1 subtypes/circulating recombinant form (CRFs) and genetic clustering with late HIV diagnosis in Japan, where new infections mainly occur among young men who have sex with men (MSM) in urban areas.

**Methods:**

Anonymized data on demographics, clinical factors and HIV genetic sequences from 39.8% of people newly diagnosed with HIV in Japan were collected by the Japanese Drug Resistance HIV‐1 Surveillance Network from 2003 to 2019. Factors associated with late HIV diagnosis (defined as HIV diagnosis with a CD4 count <350 cells/μl) were identified using logistic regression. Clusters were identified by HIV‐TRACE with a genetic distance threshold of 1.5%.

**Results:**

Of the 9422 people newly diagnosed with HIV enrolled in the surveillance network between 2003 and 2019, 7752 individuals with available CD4 count at diagnosis were included. Late HIV diagnosis was observed in 5522 (71.2%) participants. The overall median CD4 count at diagnosis was 221 (IQR: 62–373) cells/μl. Variables independently associated with late HIV diagnosis included age (adjusted odds ratio [aOR] 2.21, 95% CI 1.88–2.59, ≥45 vs. ≤29 years), heterosexual transmission (aOR 1.34, 95% CI 1.11–1.62, vs. MSM), living outside of Tokyo (aOR 1.18, 95% CI 1.05–1.32), hepatitis C virus (HCV) co‐infection (aOR 1.42, 95% CI 1.01–1.98) and not belonging to a cluster (aOR 1.30, 95% CI 1.12–1.51). CRF07_BC (aOR 0.34, 95% CI 0.18–0.65, vs. subtype B) was negatively associated with late HIV diagnosis.

**Conclusions:**

In addition to demographic factors, HCV co‐infection, HIV‐1 subtypes/CRFs and not belonging to a cluster were independently associated with late HIV diagnosis in Japan. These results imply the need for public health programmes aimed at the general population, including but not limited to key populations, to encourage HIV testing.

## 1 INTRODUCTION

The advent of antiretroviral therapy (ART) has dramatically changed the prognosis of people living with human immunodeficiency virus (HIV). Early ART initiation allows a similar life expectancy to that of people living without HIV [1]. Early diagnosis and treatment, regardless of CD4 count, are recommended to reduce HIV‐associated morbidity and mortality [2]. However, in many countries, a substantial proportion of people living with HIV (PLWH) remain undiagnosed (54.0% in the United States and Canada [3], 44% in the UK [4] and 58.8% in China [5]), with diagnosis occurring when their CD4 count decreases to <350 cells/μl. Late diagnosis confers significant clinical consequences on long‐term mortality [6], increases the risk of onward transmission [7] and higher healthcare costs [8]. Late HIV diagnosis is a major concern epidemiologically, socially and for national healthcare systems.

Japan has a low prevalence of HIV, with a cumulative total of 31,385 PLWH at the end of 2019 [9, 10]. HIV prevalence is less than 0.1% among adults aged 15–49 years [9, 10]. Men who have sex with men (MSM) constitute more than 70% of newly diagnosed PLWH, and those in urban areas are most at risk for HIV infection [9, 10]. In Japan, voluntary counselling and testing (VCT) services are available at public health centres, but only 32% of people newly diagnosed with HIV were detected through VCT [10, 11]. In recent years, approximately 30% of new HIV diagnoses were identified following the onset of acquired immune deficiency syndrome (AIDS)‐defining illnesses [9, 10]. According to previous studies, the proportion of diagnosed PLWH was estimated to be 80–85% as of 2015–2017, lower than the first 90 of the UNAIDS 90‐90‐90 goals [12, 13, 14]. The national guidelines recommend early ART initiation regardless of CD4 count [15, 16]. Once PLWH are retained in care, 99.1% experience viral suppression [12]. A number of large trials have shown that widespread early testing and early treatment reduce HIV transmission [17, 18]. These data suggest that enhancing earlier HIV diagnosis and linkage to treatment should be the core strategy in controlling the HIV epidemic. Therefore, elucidating factors associated with late HIV diagnosis could facilitate effective HIV control in Japan.

Descriptive epidemiology of the national AIDS surveillance (NAS) in Japan showed that compared to individuals newly diagnosed with HIV without AIDS‐defining illnesses, those newly diagnosed with HIV and AIDS‐defining illnesses were more likely to be older, heterosexual and from non‐urban areas [10]. Besides demographics, hepatitis B or C co‐infections [19, 20], HIV‐1 subtypes [21, 22, 23] and clustering based on nucleotide sequences [24, 25] may be associated with late diagnosis, but this information is not included in the NAS. To our knowledge, no large‐scale nationwide study has revealed the association of the aforementioned factors with late HIV diagnosis.

In the present study, we conducted a large‐scale study on late HIV diagnosis in Japan to explore the association of demographics, clinical factors and viral genetic clustering with late HIV diagnosis using data from transmitted drug resistance surveillance of HIV‐1.

## 2 METHODS

### 2.1 Study setting and participants

The Japanese Drug Resistance HIV‐1 Surveillance Network has been collecting demographic, clinical and HIV‐1 *protease* and *reverse transcriptase* (*PR‐RT*) sequencing data as part of transmitted drug resistance surveillance [24, 25, 26, 27]. Since 2003, there have been more than 100 collaborating institutions, which are widely distributed across Japan. Demographic, clinical and viral sequencing data are collected anonymously from PLWH receiving care at any of these institutions, who agree to participate. Among all PLWH registered in the Japanese Drug Resistance HIV‐1 Surveillance Network, we enrolled individuals who met the following inclusion criteria: newly diagnosed with HIV between January 2003 and December 2019; available CD4 count before the end of the year following HIV diagnosis; and ART‐naïve at the time of available CD4 count.

In Japan, all new HIV diagnoses are mandatorily notified to the public health centre by physicians under the Infectious Diseases Control Law and compiled in the NAS report [9, 10]. However, the NAS did not collect CD4 counts until 2019, nor does it collect data on hepatitis B or C co‐infection or HIV‐1 sequences. Therefore, we used data from the Japanese Drug Resistance HIV‐1 Surveillance Network. Data from the NAS report were used to assess the coverage and representativeness of the study participants.

### 2.2 Definition of late diagnosis

Late diagnosis and late diagnosis with advanced HIV infection were defined as HIV diagnosis at a CD4 count <350 and <200 cells/μl, respectively [26].

### 2.3 Variables of interest

Demographic variables of interest were: year of diagnosis, sex, age at diagnosis, transmission risk, country of origin and geographic location. Clinical variables of interest included hepatitis B surface (HBs) antigen and hepatitis C virus (HCV) antibody positivity. Phylogenetic variables of interest were HIV‐1 subtype/circulating recombinant form (CRF) and belonging to a cluster. To assess whether the period of diagnosis was associated with late diagnosis, the 17‐year study period was grouped into subperiods of 2003–2008, 2009–2014 and 2015–2019 to ensure an adequate sample size in each group for statistical analysis. If more than one transmission risk factor was reported, risk categories were ranked as follows: people who inject drugs > MSM > heterosexuals. Age at diagnosis was stratified into three groups based on the interquartile range (IQR) of 25% and 75%: ≤29, 30–44 and ≥45 years.

### 2.4 Logistic regression

Logistic regression was performed to identify factors associated with late diagnosis. Odds ratios (OR) and 95% confidence intervals (CI) were calculated. The Wald test was performed, and the corresponding *p*‐values were calculated. In the univariable analysis, crude ORs were calculated independently. Variables with *p*<0.10 were included in the multivariable analysis along with factors associated with late diagnosis: sex [5, 22], age at diagnosis [19, 27], transmission risk [5, 27] and country of origin [22, 28]. A two‐tailed *p*‐value of <0.05 was considered statistically significant. Statistical analyses were performed using JMP Pro version 15.0.0 (SAS Institute Inc., Cary, NC, USA).

### 2.5 HIV‐1 subtyping and cluster designation

Nucleotide sequences from *PR‐RT* regions (HXB2: 2253–3269) were used for HIV‐1 subtyping and identifying clusters. HIV‐1 subtype/CRF was determined using the jumping profile Hidden Markov Model [29]. All subtypes and CRFs with more than 30 sequences were categorized individually, whereas less prevalent subtypes and CRFs were grouped under “Other.” To identify clusters, HIV‐TRACE was employed with a 0.015 substitutions/site genetic distance threshold and a 0.05 ambiguity fraction [30]. All available sequences (regardless of CD4 data availability) from individuals newly diagnosed from January 2003 to December 2019 (8815 sequences) were included in the cluster analysis. Clusters were defined as containing at least three individuals, while two individuals with similar *PR‐RT* sequences were defined as a pair. An individual outside of these two categories was defined as a singleton.

### 2.6 Ethics

This study was conducted in accordance with the principles of the Declaration of Helsinki and was approved by the Medical Research Ethics Committee of the National Institute of Infectious Diseases (approval no. 1098). All participants provided written informed consent for data collection and subsequent analyses unless the requirement for written informed consent for study participation was waived by an institutional review board. Data collection was anonymized.

## 3 RESULTS

### 3.1 Participant characteristics

**Figure 1:**
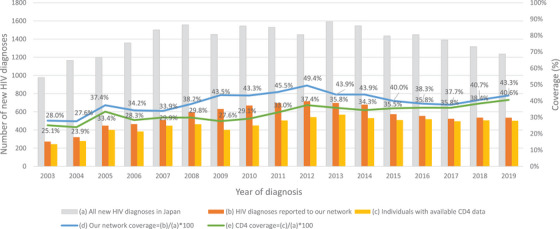
The number of new HIV diagnoses in Japan and the coverage of the Japanese Drug Resistance HIV‐1 Surveillance Network from 2003 to 2019. (a) The number of new HIV diagnoses in the National AIDS surveillance (NAS) reported from 2003 to 2019. (b) The number of new HIV diagnoses reported to the Japanese Drug Resistance HIV‐1 Surveillance Network from 2003 to 2019. (c) The number of new HIV diagnoses reported to the Japanese Drug Resistance HIV‐1 Surveillance Network with CD4 counts measured within the year following diagnosis. (d) The coverage of the Japanese Drug Resistance HIV‐1 Surveillance Network from 2003 to 2019. The number of new HIV diagnoses reported to the Network (b) was divided by the number of new HIV diagnoses reported to the NAS (a). (e) CD4 count data coverage of the Japanese Drug Resistance HIV‐1 Surveillance Network from 2003 to 2019. The number of new HIV diagnoses reported to the Network with CD4 count data measured within the year following diagnosis (c) was divided by the number of new HIV diagnoses reported to the NAS (a).

**Figure 2:**
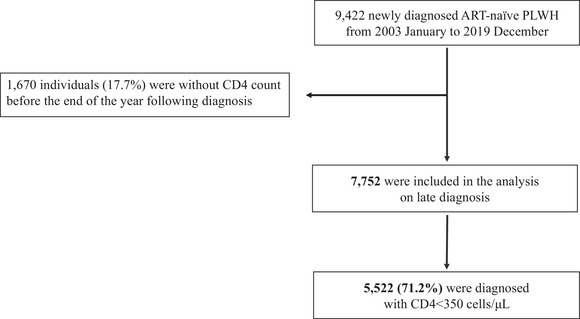
Flowchart of individuals included in the analysis and the prevalence of late HIV diagnosis. Between 2003 and 2019, 9422 newly diagnosed ART‐naïve PLWH were registered in the Japanese Drug Resistance HIV‐1 Surveillance Network. A total of 7752 individuals were included in the analysis, and 71.2% had a CD4 count <350 cells/μl at diagnosis (defined as “late diagnosis”).

From January 2003 to December 2019, our network registered 9422 newly diagnosed ART‐naïve PLWH, representing 39.8% of all newly diagnosed PLWH in Japan (*N* = 23,689) for the same period (Figure 1). The CD4 counts were unavailable for 1670 individuals (17.7%), resulting in 7752 participants being included in the analysis (Figure 2). We evaluated the representativeness of the study participants by comparing their socio‐demographic data with data from the NAS from 2003 to 2019 (*n* = 23,689) (Table S1). The characteristics of the 7752 participants were broadly similar to those of the 23,689 individuals notified to the NAS. To evaluate the potential biases caused by the unavailability of CD4 data, the characteristics of the study participants were also compared with demographic, clinical and phylogenetic data from individuals who did not have available CD4 count data (*n* = 1670) (Table S1). Among those with no available CD4 data, the percentages without data on HBV and HCV co‐infections were higher. Another difference that should be noted was the higher frequency of missing CD4 data from 2009 to 2014 compared with those from the other two periods.

Among the study participants, 5522 (71.2%) and 3569 (46.0%) had CD4 counts <350 and <200 cells/μl, respectively, at diagnosis. The median interval from HIV diagnosis to CD4 measurement was 12 (IQR 3–28) days. The characteristics of the 7752 participants are summarized based on late diagnosis CD4 categories (Table 1). The overall median CD4 count at diagnosis was 221 (IQR: 62–373) cells/μl. Most participants were male (95.1%) and Japanese (90.9%). The median age at HIV diagnosis was 36 (IQR: 30–45) years. The predominant transmission risk was MSM (76.8%), followed by heterosexual (16.7%) and injecting drug use (0.8%). The prevalence of HCV antibody seropositivity was 3.3% (256/7752 participants) at HIV diagnosis. Among 256 individuals with HCV co‐infection, 182 (71.1%) were MSM, 33 (12.9%) were heterosexuals, 25 (9.8%) were other/unreported and 16 (6.3%) were people who inject drugs. Among 60 people who inject drugs, 16 (26.6%) were HCV seropositive, which is higher than the HCV antibody seropositivity of the overall participants (256/7752; 3.3%).

**Table 1:** Characteristics of the study participants according to the CD4 count (cells/μl) at HIV diagnosis in Japan, 2003–2019.

	ALL		CD4 count (cells/μl) at diagnosis	
Characteristics	*n* (%)	Median CD4 count at diagnosis [IQR]	<350 *n* (%)	<200 *n* (%)
**All**	7752 (100.0%)	221 [62–373]	5522 (71.2%)	3569 (46.0%)
**Period of diagnosis**				
2003–2008	2221 (28.7%)	229 [67–376]	1559 (70.2%)	1000 (45.0%)
2009–2014	2998 (38.7%)	210 [54–360]	2190 (73.0%)	1428 (47.6%)
2015–2019	2533 (32.7%)	229 [67–381]	1773 (70.0%)	1141 (45.0%)
**Sex**				
Male	7375 (95.1%)	221 [62–373]	5255 (71.3%)	3389 (46.0%)
Female	370 (4.8%)	214 [54–379]	261 (70.5%)	175 (47.3%)
Unknown	7 (0.1%)	44 [15–212]	6 (85.7%)	5 (71.4%)
**Age (years)**				
≤29	1972 (25.4%)	296 [182–424]	1185 (60.1%)	559 (28.3%)
30–44	3789 (48.9%)	219 [62–371]	2709 (71.5%)	1765 (46.6%)
≥45	1928 (24.9%)	125 [32–300]	1582 (82.1%)	1213 (62.9%)
Unknown	63 (0.8%)	186 [40–354]	46 (73.0%)	32 (50.8%)
**Transmission risk**				
MSM	5956 (76.8%)	240 [79–386]	4126 (69.3%)	2526 (42.4%)
Heterosexual^a^	1297 (16.7%)	162 [33–338]	994 (76.6%)	727 (56.1%)
Male	971 (12.5%)	144 [29–314]	765 (78.8%)	575 (59.2%)
Female	324 (4.2%)	230 [57–380]	227 (70.1%)	150 (46.3%)
PWID	60 (0.8%)	164 [26–401]	43 (71.7%)	32 (53.3%)
Other/unreported	439 (5.7%)	80 [24–285]	359 (81.8%)	284 (64.7%)
**Country of origin**				
Japan	7045 (90.9%)	221 [62–372]	5027 (71.4%)	3251 (46.1%)
Other	683 (8.8%)	221 [63–379]	481 (70.4%)	306 (44.8%)
Unknown	24 (0.3%)	237 [62–450]	14 (58.3%)	12 (50.0%)
**Geographical area**				
Tokyo	2890 (37.3%)	242 [90–388]	1972 (68.2%)	1211 (41.9%)
Other areas	4862 (62.7%)	208 [49–363]	3550 (73.0%)	2358 (48.5%)
**HBs antigen**				
Positive	571 (7.4%)	202 [61–351]	427 (74.8%)	280 (49.0%)
Negative	6510 (84.0%)	222 [62–373]	4644 (71.3%)	2987 (45.9%)
Unknown	671 (8.7%)	234 [68–413]	451 (67.2%)	302 (45.0%)
**HCV antibody**				
Positive	256 (3.3%)	161 [47–313]	204 (79.7%)	143 (55.9%)
Negative	6763 (87.2%)	222 [62–374]	4813 (71.2%)	3094 (45.7%)
Unknown	733 (9.5%)	231 [67–393]	505 (68.9%)	332 (45.3%)
**HIV‐1 subtype/CRF**				
B	6416 (82.8%)	220 [64–371]	4595 (71.6%)	2965 (46.2%)
CRF01_AE	559 (7.2%)	176 [24–352]	418 (74.8%)	290 (51.9%)
C	100 (1.3%)	205 [65–349]	75 (75.0%)	50 (50.0%)
CRF02_AG or G	94 (1.2%)	185 [35–341]	71 (75.5%)	50 (53.2%)
CRF07_BC	44 (0.6%)	376 [236–465]	18 (40.9%)	5 (11.4%)
A	35 (0.5%)	186 [46–392]	25 (71.4%)	18 (51.4%)
Other	74 (1.0%)	298 [194–461]	44 (59.5%)	19 (25.7%)
Unavailable	430 (5.5%)	284 [81–434]	276 (64.2%)	172 (40.0%)
**Cluster category**				
Clustered	5174 (66.7%)	229 [69–376]	3642 (70.4%)	2309 (44.6%)
Pair	439 (5.7%)	236 [85–378]	303 (69.0%)	187 (42.6%)
Singleton	1709 (22.0%)	182 [41–339]	1301 (76.1%)	901 (52.7%)
Unavailable	430 (5.5%)	284 [81–434]	276 (64.2%)	172 (40.0%)

Abbreviations: IQR, interquartile range; MSM, men who have sex with men; PWID, people who inject drugs.

^a^ There were two individuals of unspecified sex.

### 3.2 Late diagnosis trends over time

**Figure 3:**
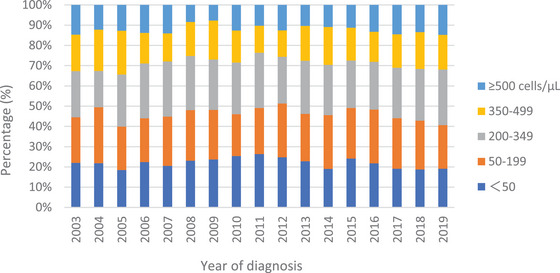
CD4 count at HIV diagnosis of 7752 individuals from 2003 to 2019: Japanese Drug Resistance HIV‐1 Surveillance Network. Percentages of CD4 count at HIV diagnosis: <50 cells/μl (blue), 50–199 cells/μl (orange), 200–349 cells/μl (grey), 350–499 cells/μl (yellow) and ≥500 cells/μl (sky blue) are shown by year of diagnosis.

Figure 3 shows the annual CD4 distributions grouped into the following five CD4 count categories: <50, 50–199, 200–349, 350–499 and ≥500 cells/μl. The distribution of CD4 counts <350 and <200 cells/μl at HIV diagnosis according to the year of diagnosis was approximately 70% and 40–50%, respectively, with minor fluctuations over the 17‐year period. During 2003–2008, 70.2% of new HIV diagnoses were late diagnoses, and the proportion increased to 73.0% in 2009–2014 before decreasing to 70.0% in 2015–2019 (Table 1).

### 3.3 HIV‐1 subtyping and cluster category

Subtype B accounted for 82.8%, followed by CRF01_AE (7.2%). Other subtypes or CRFs comprising more than 30 sequences included subtype C, CRF02_AG or subtype G, CRF07_BC, and subtype A. Two hundred and eighty‐four clusters and 273 pairs were identified. The median number of individuals in a cluster was 5 (range, 3–2533). Among the individuals with an available CD4 count, 5174 (67%) were in clusters, 439 (5.7%) were in pairs and 1709 (22.0%) were singletons. The median CD4 counts [IQR] (cells/μl) for clusters, pairs and singletons were 229 [69–376], 236 [85–378] and 182 [41–339], respectively.

### 3.4 Factors associated with late diagnosis

The results of the logistic regression analysis are shown in Table 2. In a univariable model, the late diagnosis was associated with the period of diagnosis (2009–2014 vs. 2015–2019), higher age (≥45 and 30–44 vs. ≤29 years), transmission risk (heterosexual vs. MSM), location (other vs. Tokyo), HCV antibody seropositivity and singleton (vs. clustered). HIV CRF07_BC and other subtypes were inversely associated with late diagnosis (vs. subtype B). A potential association between late HIV diagnosis and HBs antigen positivity (*p*<0.10) was identified. All variables of interest were included in the multivariable model, and factors associated with late diagnosis were higher age (≥45: 2.21 [1.88–2.59] and 30–44: 1.49 [1.31–1.69] vs. ≤29 years), transmission risk (heterosexual: 1.34 [1.11–1.62] vs. MSM), location (other: 1.18 [1.05–1.32] vs. Tokyo), HCV antibody positivity (1.42 [1.01–1.98] vs. negative) and singleton (1.30 [1.12–1.51] vs. clustered). HIV‐1 CRF07_BC (0.34 [0.18–0.65]) and other (0.54 [0.32–0.91] vs. subtype B) were associated with a lower risk of late diagnosis. The results were similar for late diagnosis with advanced HIV infection (Table S2).

**Table 2:** Factors associated with late HIV diagnosis (CD4 <350 cells/μl) in Japan, 2003–2019.

		Univariable analysis			Multivariable analysis		
Variables		Crude OR	95% CI	*p*‐value^a^	aOR^b^	95% CI	*p*‐value^a^
**Period of diagnosis**	2015–2019	ref			ref		
	2009–2014	1.16	1.03–1.31	0.01	1.10	0.97–1.25	0.14
	2003–2008	1.01	0.89–1.14	0.88	0.99	0.86–1.14	0.92
**Sex**	Male	ref			ref		
	Female	0.97	0.77–1.21	0.77	0.83	0.60–1.16	0.28
**Age group (years)**	≤29	ref			ref		
	30–44	1.57	1.40–1.77	<0.0001	**1.49**	1.31–1.69	<0.0001
	≥45	2.54	2.20–2.94	<0.0001	**2.21**	1.88–2.59	<0.0001
**Transmission risk**	MSM	ref			ref		
	Heterosexual	1.46	1.27–1.67	<0.0001	**1.34**	1.11–1.62	0.002
	PWID	1.12	0.64–1.97	0.69	1.34	0.65–2.78	0.42
	Other/unreported	1.99	1.55–2.55	<0.0001	**2.07**	1.53–2.81	<0.0001
**Country of origin**	Japan	ref			ref		
	Other	0.96	0.80–1.14	0.61	0.97	0.78–1.21	0.79
**Geographical area**	Tokyo	ref			ref		
	Other areas	1.26	1.14–1.39	<0.0001	**1.18**	1.05–1.32	0.006
**HBs antigen**	Negative	ref			ref		
	Positive	1.19	0.98–1.45	0.08	1.20	0.97–1.49	0.09
**HCV antibody**	Negative	ref			ref		
	Positive	1.59	1.17–2.16	0.003	**1.42**	1.01–1.98	0.04
**HIV‐1 subtype/CRF**	B	ref			ref		
	CRF01_AE	1.17	0.96–1.43	0.11	1.00	0.79–1.27	0.99
	C	1.19	0.75–1.88	0.46	0.99	0.58–1.68	0.97
	CRF02_AG or G	1.22	0.76–1.96	0.40	0.93	0.53–1.62	0.80
	CRF07_BC	0.27	0.15–0.50	<0.0001	**0.34**	0.18–0.65	0.001
	A	0.99	0.47–2.07	0.98	0.80	0.32–2.01	0.64
	Other	0.58	0.36–0.93	0.02	**0.54**	0.32–0.91	0.02
**Cluster category**	Clustered	ref			ref		
	Pair	0.94	0.76–1.16	0.55	1.04	0.82–1.31	0.78
	Singleton	1.34	1.18–1.52	<0.0001	**1.30**	1.12–1.51	0.001

Abbreviations: aOR, adjusted odds ratio; CI, confidence interval; MSM, men who have sex with men; OR, odds ratio; PWID, people who inject drugs.

^a^ *p*‐values were calculated using the Wald test. A two‐tailed *p*<0.05 was considered statistically significant.

^b^ Statistically significant adjusted odds ratios are shown in bold.

## 4 DISCUSSION

This is the first nationwide study of late HIV diagnosis in Japan. A strength of our study is the large number of individuals recruited from the national surveillance network over the 17‐year study period, which facilitated the analysis of the distribution of CD4 counts at the time of diagnosis. The majority (71.2%) of PLWH were diagnosed with a CD4 count <350 cells/μl, and approximately half (46.0%) were diagnosed with a CD4 count <200 cells/μl, which is much higher than previously reported for the UK, Australia, North America and China (44% and 23% [4], 39% and 20% [31], 54% and unavailable [3], and 59% and 35% [5], respectively). The analysis by risk group showed the same trend: 69.3% of MSM had a CD4 count <350 cells/μl at HIV diagnosis, which was much higher than that reported in a previous study among Chinese MSM (43.9%) [5]. An increasing trend towards early diagnosis was reported in studies from the United States and Canada [3], the Netherlands [27], Europe [32] and China [5]. We investigated whether late diagnosis in Japan decreased during the 17‐year period. Although the proportion of late HIV diagnoses showed a slight decrease from 73% in 2009–2014 to 70% in 2015–2019, proportions remained high throughout the study period compared with those in other countries. Thus, there is an urgent need for public health measures to diagnose HIV infection earlier.

Our analysis showed that older age and identifying as heterosexual were independent risk factors for late diagnosis, which is consistent with the results of studies in other countries [5, 22]. Individuals older than 50 years and heterosexuals tend to believe that they are not at risk for HIV infection [33]. The results suggest that HIV testing focusing only on key populations is insufficient to reduce the high proportion of late diagnoses.

Regarding the regional effect on HIV diagnosis, a remarkable difference in the risk of late diagnosis between Tokyo and other areas was identified. Tokyo is the largest city in Japan, and 37.3% of all new HIV diagnoses in Japan between 2003 and 2019 were reported in Tokyo [10]. A similar trend for rural residents to be diagnosed later than that observed in individuals residing in large cities has been reported [28]. The difference between Tokyo and other areas was possibly caused by several factors, including HIV‐related stigma and discrimination, insufficient HIV awareness and lack of access to facilities with HIV‐related expertise [34]. Although our analysis showed a regional difference within Japan, the proportion of late diagnoses even in Tokyo was 68.2%, which was higher than the aforementioned studies from other countries in which study populations were not limited to urban areas. This study suggests that late diagnosis is a national problem.

After adjusting for other factors, HCV, but not HBV, was significantly associated with late HIV diagnosis, as previously reported [19, 20]. Injecting drug use is a major risk factor for HCV infection [35]. Despite the high prevalence of HCV antibody, injecting drug use was not associated with late diagnosis, whereas HCV positivity and late HIV diagnosis remained significantly associated after adjusting for transmission risk. While HCV is believed to be transmitted by unprotected anal intercourse among MSM [36], HCV infection is rare in HIV‐negative MSM [37]. There are two possible reasons: HIV infection biologically increases the risk of HCV infection [38], and HIV is more easily transmitted by sexual contact than HCV [39]. Furthermore, HIV infection often precedes HCV infection in MSM [39, 40]. Therefore, many HCV antibody‐positive participants at the time of HIV diagnosis likely acquired HCV following HIV infection and before HIV diagnosis, possibly related to a long period of high‐risk behaviour, that increases the risk of HCV infection. This assumption partly explains the association between HCV antibody positivity and late HIV diagnosis. Another possible explanation is that HCV co‐infection may affect the CD4 count, which decreases after HCV seroconversion in PLWH compared with PLWH only [40]. However, the effect of HCV co‐infection on reduced CD4 count is transient [36], and HCV serostatus does not affect HIV disease progression [41]. Taken together, multiple factors, such as the temporal sequence of HIV and HCV acquisition, time to HCV acquisition and impact of HCV on CD4 count, could have contributed to the association between HCV and late HIV diagnosis. One important caveat is that the association between HCV and late HIV diagnosis was evaluated using HCV antibody positivity, which indicates a history of infection but does not necessarily indicate current infection.

CRF07_BC showed a negative association with late diagnosis. CRF07_BC was first reported in 1997 in China [42]. CRF07_BC infection was first identified in Japan in 2006, with only two individuals being reported until 2012. However, the number of reports increased to 41 between 2013 and 2019. The negative association suggests that late diagnosis is less common in subtypes recently introduced than in those prevalent in Japan for longer. HIV‐1 CRF07_BC has been associated with slower immunological progression compared to HIV‐1 subtype B in previous studies, which may be due to the 7‐amino acid deletion in p6 of CRF07_BC [43, 44]. Given this, the possibility that virological differences between subtypes might have contributed to the result of this study cannot be excluded.

The sequence analysis showed that singletons were significantly associated with late HIV diagnosis. This may be due to the virus evolving within the individual beyond the threshold of genetic distance over a long period of time [45], or the virus may have been transmitted from undiagnosed individuals [46]. We employed a 1.5% threshold, which corresponds to a maximum of approximately 7–8 years of viral evolution [47], to minimize the influence of intra‐patient viral evolution on clustering. One hypothesis for the association between singletons and late diagnosis is that individuals with a partner or partners with HIV could have high‐risk perception and actively seek HIV testing, as seen in previous epidemiological studies [48, 49]. In this study, a similar effect was observed. Given that singletons would be missed by partner services or programmes based on clusters [50], different approaches may be needed to address late diagnosis.

Our study found that older age, heterosexual transmission, attending medical institutions outside of Tokyo, HCV positivity and not being in a viral *PR‐RT* sequence cluster are associated with late HIV diagnosis. Individuals who are older, heterosexual and attend medical institutions outside of Tokyo have different characteristics from key populations in Japan and are usually excluded from priority programmes focusing on key populations. Thus, there is a further need to expand HIV programmes that currently focus on key populations for a broader population. In Japan, there is low awareness of free anonymous VCT services conducted by local governments [51, 52], and in some areas outside of large cities, VCT services are infrequent [53]. Additionally, HIV self‐testing has not been officially approved in Japan [11]. Strategies that focus on key populations remain very important for early HIV diagnosis, but facilitating access to testing, including VCT services and self‐testing, for a broader population is critical to reducing the high occurrence of late diagnoses and achieving 95‐95‐95 targets in Japan.

Several limitations of this study should be considered. First, the study group covered only 39.8% of individuals who were diagnosed with HIV between 2003 and 2019 in Japan, of whom 82% had available data on CD4 count at diagnosis. While there were no major differences in socio‐demographic characteristics between the study participants and all individuals newly diagnosed with HIV notified to the NAS, the percentage of MSM was higher among the study participants. This might be due to differences in the place, timing and method of collecting information; while NAS is based on data collected at the time of diagnosis from where individuals are diagnosed, our network collects data from medical institutions where individuals are followed after diagnosis. This difference might have contributed to the higher percentage of MSM and the lower percentage of individuals in the others/unreported risk category among the study participants. Therefore, we hypothesize that this difference might reflect our data on transmission risk being more detailed than the NAS and should not significantly impact the study results. Also, possible biases resulting from the unavailability of CD4 data should be considered. The higher percentages of missing data on HBV and HCV co‐infections in individuals without an available CD4 count could have influenced the impact of hepatitis co‐infections on late HIV diagnosis. A higher frequency of missing CD4 data for the 2009–2014 period may also have contributed to the slightly higher proportion of late HIV diagnoses in 2009–2014. Second, we used the available CD4 count within the year following HIV diagnosis rather than the CD4 count at diagnosis because only the year of diagnosis was available for some individuals. Therefore, the proportion of late diagnoses might have been overestimated. However, the percentage of individuals with a CD4 count <350 cells/μl at diagnosis was similar to data collected by the NAS in 2019. In 2019, the percentage was 68.1% in our study compared to 70.9% reported by the NAS. These data suggest that possible biases in the study population and the timing of CD4 count measurement did not significantly affect the results of this study. Third, clusters identified in this study may not necessarily represent the actual transmission network. A cluster may be a proxy for engagement with HIV testing services and/or healthcare in general, and there may be other larger or unidentified networks that might have been missed due to incomplete sampling.

## 5 CONCLUSIONS

In addition to demographic factors, HCV co‐infection, HIV‐1 subtypes/CRFs and not belonging to a cluster were independently associated with late HIV diagnosis in Japan. These results imply the need for public health programmes aimed at the general population, regardless of risk group, to encourage HIV testing.

## COMPETING INTERESTS

DW received honoraria from Gilead Sciences, K.K. ViiV Healthcare K.K. MSD K.K. Janssen Pharmaceutical K.K. All other authors declare no competing interests.

## AUTHORS’ CONTRIBUTIONS

MO, TK, TS and TM conceived and designed the analyses. All authors were responsible for data collection and management. MO and TK performed the statistical analyses. MO wrote the manuscript and generated tables and figures. All authors critically reviewed and approved the final version of the manuscript.

## FUNDING

This work was supported by the Japan Agency for Medical Research and Development (AMED) [grant numbers JP18fk0410005, JP21fk0410028, JP21fk0410035 and JP22fk0410050]. The funders had no role in the study design, data collection and analysis, decision to publish or manuscript preparation.

## Supporting information


**Table S1**. Characteristics of all individuals newly diagnosed with HIV notified to the National AIDS Surveillance (NAS) in 2003–2019, study participants, and individuals excluded from the analysis due to CD4 data unavailability.

**Table S2**. Factors associated with late diagnosis with advanced HIV infection (CD4 <200 cells/µL) in Japan, 2003–2019.

## Data Availability

Data for this study cannot be posted in a supplemental file or a public repository because of legal and ethical restrictions.
