# Supplementary material for: Association of demographics, HCV co‐infection, HIV‐1 subtypes and genetic clustering with late HIV diagnosis: a retrospective analysis from the Japanese Drug Resistance HIV‐1 Surveillance Network
Source: J Int AIDS Soc. 2023 May 23;26(5):e26086. doi: 10.1002/jia2.26086 (PMC10206413; doi:10.1002/jia2.26086)
Supplement: Supplementary file 1 — Table S1. Characteristics of all individuals newly diagnosed with HIV notified to the National AIDS Surveillance (NAS) in 2003–2019, study participants, and individuals excluded from the analysis due to CD4 data unavailability. [file JIA2-26-e26086-s002.docx]

**Table S1. Characteristics of all individuals newly diagnosed with HIV notified to the National AIDS Surveillance (NAS) in 2003–2019, study participants, and individuals excluded from the analysis due to CD4 data unavailability.**

| **Characteristics** | **All individuals newly diagnosed with HIV** | **Study participants** | **Individuals**  **without CD4 data** |
| --- | --- | --- | --- |
| **All** | 23,689 | 7,752 | 1,670 |
| **Period of diagnosis** |  |  |  |
| 2003–2008 | 7,755 (32.7%) | 2,221 (28.7%) | 389 (23.3%) |
| 2009–2014 | 9,110 (38.5%) | 2,998 (38.7%) | 1,091 (65.3%) |
| 2015–2019 | 6,824 (28.8%) | 2,533 (32.7%) | 190 (11.4%) |
| **Gender** |  |  |  |
| Male | 22,192 (93.7%) | 7,375 (95.1%) | 1,571 (94.1%) |
| Female | 1,497 (6.3%) | 370 (4.8%) | 96 (5.7%) |
| Unknown | 0 (0.0%) | 7 (0.1%) | 3 (0.2%) |
| **Age (years)** |  |  |  |
| ≤29 | 6,130 (25.9%) | 1,972 (25.4%) | 313 (18.7%) |
| 30–44 | 11,281 (47.6%) | 3,789 (48.9%) | 803 (48.1%) |
| ≥45 | 6,273 (26.4%) | 1,928 (24.9%) | 483 (28.9%) |
| Unknown | 5 (0.0%) | 63 (0.8%) | 71 (4.3%) |
| **Transmission risk** |  |  |  |
| MSM | 14,822 (62.6%) | 5,956 (76.8%) | 1,041 (62.3%) |
| Heterosexuals | 5,244 (22.1%) | 1,297 (16.7%)^†^ | 269 (16.1%)^‡^ |
| Male | 4,186 (17.7%) | 971 (12.5%) | 183 (11.0%) |
| Female | 1,058 (4.5%) | 324 (4.2%) | 85 (5.1%) |
| PWID | 100 (0.4%) | 60 (0.8%) | 2 (0.1%) |
| Others/unreported | 3,523 (14.9%) | 439 (5.7%) | 358 (21.4%) |
| **Country of origin** |  |  |  |
| Japan | 21,111 (89.1%) | 7,045 (90.9%) | 1,513 (90.6%) |
| Others | 978 (4.1%) | 683 (8.8%) | 142 (8.5%) |
| Unknown | 1,600 (6.8%) | 24 (0.3%) | 15 (0.9%) |
| **Geographical area** |  |  |  |
| Tokyo | 7,707 (32.5%) | 2,890 (37.3%) | 508 (30.4%) |
| Other areas | 15,982 (67.5%) | 4,862 (62.7%) | 1,162 (69.6%) |
| **HBs antigen** |  |  |  |
| Positive | N/A | 571 (7.4%) | 76 (4.6%) |
| Negative | N/A | 6,510 (84.0%) | 883 (52.9%) |
| Unknown | N/A | 671 (8.7%) | 711 (42.6%) |
| **HCV antibody** |  |  |  |
| Positive | N/A | 256 (3.3%) | 39 (2.3%) |
| Negative | N/A | 6,763 (87.2%) | 823 (49.3%) |
| Unknown | N/A | 733 (9.5%) | 808 (48.4%) |
| **HIV–1 subtype/CRF** |  |  |  |
| B | N/A | 6,416 (82.8%) | 1,316 (78.8%) |
| CRF01_AE | N/A | 559 (7.2%) | 131 (7.8%) |
| C | N/A | 100 (1.3%) | 14 (0.8%) |
| CRF02_AG or G | N/A | 94 (1.2%) | 13 (0.8%) |
| CRF07_BC | N/A | 44 0.6%) | 5 (0.3%) |
| A | N/A | 35 (0.5%) | 3 (0.2%) |
| Others | N/A | 74 (1.0%) | 11 (0.7%) |
| Unavailable | N/A | 430 (5.5%) | 177 (10.6%) |
| **Cluster category** |  |  |  |
| Clustered | N/A | 5,174 (66.7%) | 1,070 (64.1%) |
| Pair | N/A | 439 (5.7%) | 107 (6.4%) |
| Singleton | N/A | 1,709 (22.0%) | 316 (18.9%) |
| Unavailable | N/A | 430 (5.5%) | 177 (10.6%) |

ABBREVIATIONS: MSM, men who have sex with men; PWID, people who inject drugs; N/A, not available.

†Two individuals of unspecified sex.

‡One individual of unspecified sex.
