# Supplementary material for: Association of demographics, HCV co‐infection, HIV‐1 subtypes and genetic clustering with late HIV diagnosis: a retrospective analysis from the Japanese Drug Resistance HIV‐1 Surveillance Network
Source: J Int AIDS Soc. 2023 May 23;26(5):e26086. doi: 10.1002/jia2.26086 (PMC10206413; doi:10.1002/jia2.26086)
Supplement: Supplementary file 2 — Table S2. Factors associated with late diagnosis with advanced HIV infection (CD4 <200 cells/µL) in Japan, 2003–2019. [file JIA2-26-e26086-s001.docx]

**Table S2. Factors associated with late diagnosis with advanced HIV infection (CD4 <200 cells/μL) in Japan, 2003–2019**

|  |  | **Univariable analysis** | | | **Multivariable analysis** | | |
| --- | --- | --- | --- | --- | --- | --- | --- |
| **Variables** |  | **Crude OR** | **95%CI** | **p-value^†^** | **aOR^‡^** | **95%CI** | **p-value^†^** |
| **Period of diagnosis** | 2015–2019 | ref |  |  | ref |  |  |
|  | 2009–2014 | 1.11 | 0.99 to 1.23 | 0.05 | 1.02 | 0.91 to 1.15 | 0.74 |
|  | 2003–2008 | 1.00 | 0.89 to 1.12 | 0.99 | 0.95 | 0.83 to 1.08 | 0.43 |
| **Sex** | Male | ref |  |  | ref |  |  |
|  | Female | 1.06 | 0.86 to 1.30 | 0.61 | 0.78 | 0.59 to 1.05 | 0.10 |
| **Age group (years)** | ≤29 | ref |  |  | ref |  |  |
|  | 30–44 | 2.14 | 1.90 to 2.40 | <0.0001 | **1.98** | 1.74 to 2.25 | <0.0001 |
|  | ≥45 | 3.91 | 3.42 to 4.47 | <0.0001 | **3.31** | 2.86 to 3.84 | <0.0001 |
| **Transmission risk** | MSM | ref |  |  | ref |  |  |
|  | Heterosexuals | 1.73 | 1.53 to 1.95 | <0.0001 | **1.58** | 1.35 to 1.86 | <0.0001 |
|  | PWID | 1.55 | 0.93 to 2.58 | 0.09 | 1.59 | 0.86 to 2.96 | 0.14 |
|  | Others/unreported  unreported | 2.49 | 2.03 to 3.05 | <0.0001 | **2.41** | 1.89 to 3.07 | <0.0001 |
| **Country of origin** | Japan | ref |  |  | ref |  |  |
|  | Others | 0.95 | 0.81 to 1.11 | 0.50 | 0.98 | 0.80 to 1.20 | 0.81 |
| **Geographical area** | Tokyo | ref |  |  | ref |  |  |
|  | Other areas | 1.31 | 1.19 to 1.43 | < 0.0001 | **1.22** | 1.10 to 1.36 | 0.0003 |
| **HBs antigen** | Negative | ref |  |  |  | N/A^§^ |  |
|  | Positive | 1.13 | 0.96 to 1.35 | 0.15 |  |  |  |
| **HCV antibody** | Negative | ref |  |  | ref |  |  |
|  | Positive | 1.50 | 1.17 to 1.93 | 0.002 | 1.21 | 0.92 to 1.59 | 0.17 |
| **HIV–1 subtype/CRF** | B | ref |  |  | ref |  |  |
|  | CRF01_AE | 1.25 | 1.06 to 1.49 | 0.01 | 1.01 | 0.82 to 1.25 | 0.93 |
|  | C | 1.16 | 0.78 to 1.73 | 0.45 | 0.86 | 0.54 to 1.35 | 0.51 |
|  | CRF02_AG/G | 1.32 | 0.88 to 1.99 | 0.18 | 0.94 | 0.58 to 1.52 | 0.80 |
|  | CRF07_BC | 0.15 | 0.06 to 0.38 | <0.0001 | **0.19** | 0.07 to 0.50 | 0.0008 |
|  | A | 1.23 | 0.63 to 2.40 | 0.54 | 0.83 | 0.36 to 1.89 | 0.65 |
|  | Others | 0.40 | 0.24 to 0.68 | 0.0007 | **0.34** | 0.19 to 0.61 | 0.0003 |
| **Cluster category** | Clustered | ref |  |  | ref |  |  |
|  | Pair | 0.92 | 0.76 to 1.12 | 0.41 | 0.96 | 0.77 to 1.20 | 0.72 |
|  | Singleton | 1.38 | 1.24 to 1.54 | < 0.0001 | **1.32** | 1.15 to 1.50 | <0.0001 |

ABBREVIATIONS: OR, odds ratio; aOR, adjusted odds ratio; CI, confidence interval; MSM, men who have sex with men; PWID, people who inject drugs; N/A, not available.

^†^ *P*–values were calculated using the Wald test. A two–tailed *p*<0.05 was considered statistically significant.

^‡^Statistically significant adjusted odds ratios are shown in bold.

^§^HBs antigen was not included in the multivariate analysis because the *p*–value from the univariable analysis was >0.10.
